# Burden trends and future predictions for hypertensive heart disease attributable to non-optimal temperatures in the older adults amidst climate change, 1990–2021

**DOI:** 10.3389/fpubh.2024.1525357

**Published:** 2025-01-03

**Authors:** Can Xu, Xinyu Nie, Rui Xu, Ge Han, Dongjin Wang

**Affiliations:** ^1^Department of Cardiac Surgery, Nanjing Drum Tower Hospital, The Affiliated Hospital of Nanjing University Medical School, Nanjing, China; ^2^Nanjing University Medical School, Nanjing, China; ^3^The Second Hospital of Nanjing, Nanjing University of Chinese Medicine, Nanjing, China

**Keywords:** hypertensive heart disease, global disease burden, older adults population, non-optimal temperature, low temperature, high temperature, trend

## Abstract

**Background:**

Hypertensive heart disease (HHD) is a significant form of end-organ damage caused by hypertension, with profound impacts on global health and quality of life. Temperature anomalies driven by climate change, particularly extremes of heat and cold, are increasingly recognized as major contributors to the cardiovascular disease burden, notably impacting HHD. However, the specific spatiotemporal trends and gender-based differences in the burden of non-optimal temperatures on older adults HHD patients remain insufficiently explored. This study aims to evaluate the regional, gender-specific trends in the burden of HHD attributed to non-optimal temperatures among the older adults from 1990 to 2021, and to project future trends in HHD burden under climate-induced temperature anomalies from 2022 to 2050.

**Methods:**

Data were sourced from the Global Burden of Disease Study (GBD 2021), which provides estimates of mortality and disability-adjusted life years (DALYs) at global, regional, and national levels. Age-standardized rates (ASR) and estimated annual percentage changes (EAPC) were analyzed. Future burden projections were modeled using age-period-cohort (APC) and Bayesian APC models to assess temperature impact by gender and age differences. Data analysis was conducted using R and STATA, examining the variations in temperature effects by gender and age.

**Results:**

Between 1990 and 2021, cold-related HHD burden among the older adults significantly exceeded that of heat-related burden. However, heat-related HHD burden demonstrated a marked upward trend, projected to continue rising over the next two decades, particularly in low-income and tropical regions. Gender-specific analysis revealed that cold-related HHD burden was more pronounced in women, while heat-related burden was notably higher in men. Additionally, male heat-related HHD mortality rates have shown a substantial increase over the past 30 years, whereas female rates have exhibited a comparatively modest decline.

**Conclusion:**

Although cold remains the dominant non-optimal temperature factor, rising global temperatures suggest an increasing burden of heat-related HHD among the older adults. Efforts should prioritize strengthening resilience in vulnerable regions and populations, with targeted interventions to mitigate future health risks associated with temperature extremes.

## 1 Introduction

Hypertensive heart disease (HHD) is a type of end-organ damage caused by hypertension, often accompanied by a series of adaptive changes in the heart and blood vessels, and is closely associated with cardiovascular diseases such as heart failure and ischemic heart disease. It has a profound impact on global health and quality of life (1, 2). The definition of HHD includes left ventricular hypertrophy, cardiac dysfunction (including both systolic and diastolic dysfunction), and vascular changes. Its clinical manifestations are diverse, ranging from left ventricular hypertrophy, left atrial enlargement, and atrial fibrillation to heart failure (both reduced and preserved ejection fraction), vascular damage, and even ischemic heart disease (3, 4). Studies show that approximately one-quarter of heart failure cases are caused by HHD (5, 6), and patients with HHD face a significantly higher risk of cardiovascular events (such as myocardial infarction, congestive heart failure, stroke, and sudden death) compared to individuals with simple hypertension. As the global aging process accelerates, the proportion of older adults populations is rising significantly—9.2% of the global population was aged 60 and above in 1990; this increased to 11.7% by 2013 and is projected to reach 21.1% by 2050 (7). As individuals age, the cumulative damage caused by long-term hypertension to the heart and blood vessels leads to changes in cardiac structure and function, such as left ventricular hypertrophy and diastolic dysfunction, which are typical manifestations of HHD (8). Therefore, HHD is a condition highly associated with age and is expected to impose a heavier health burden on the older adults population (9).

In recent years, due to the sharp increase in greenhouse gas emissions, temperature anomalies have become a major global public health threat (10). The Lancet Countdown to Climate Change points out that climate change could be the greatest health threat of the 21st century (11). Since 1981, the global annual average temperature has risen at a rate of approximately 0.18°C per decade. In 2019, the global land and ocean surface temperature rose by 0.95°C above the historical average, making it the second hottest year on record (12). These changes suggest that frequent heatwaves may become unavoidable in the future. Epidemiological studies indicate that temperature anomalies are a major risk factor for cardiovascular disease, closely associated with cause-specific mortality and morbidity (13–15). In recent years, extreme heatwaves and cold spells due to climate change have become increasingly frequent worldwide. Temperature anomalies and HHD pose serious challenges to global public health, yet there is a lack of systematic analysis on the burden of HHD due to temperature anomalies and its trends. Raising public awareness of the risks of temperature anomalies on HHD, particularly in one of the vulnerable populations, the older adults, has become a global priority.

This study aims to evaluate the regional distribution, etiology, and gender-specific trends of HHD burden caused by non-optimal temperatures among the older adults from 1990 to 2021, and to project gender-specific changes in this burden from 2022 to 2050. Our findings provide crucial evidence for health policy formulation, supporting countries and temperature-sensitive populations in tackling current temperature-related health challenges and strengthening resilience to future environmental risks.

## 2 Methods

### 2.1 Data sources

The data is sourced from GBD 2021, which provides systematic estimates of mortality and disability-adjusted life years (DALYs) at global, regional, and national levels for the period 1990–2021, categorized by cause, sex, and age, and specifically analyzes the attributable effects of 87 risk factors. The methodology has been elaborated in previous studies (16, 17). GBD divides the world into 21 geographic regions, analyzing age-standardized rates (ASR) and the estimated annual percentage change (EAPC) to observe trends in mortality and DALYs caused by non-optimal temperatures for HHD. The calculation of age-standardization requires additional standard population data.^1^ To eliminate the influence of demographic structure differences, the standard population structure is based on the GBD world population age distribution (18). The dynamic changes in ASR more effectively reflect the trend of disease changes within populations, thereby providing a basis for formulating preventive strategies for HHD. EAPC is used to quantify the trend of ASR changes among different groups over a specific period. The Sociodemographic Index (SDI) is used to comprehensively assess the socio-economic and demographic development levels of each country and region, based on indicators such as GDP per capita, education level, and total fertility rate, with SDI values ranging from 0 to 1, where a higher value indicates a higher level of socio-economic development (19). Based on SDI values, 204 countries and regions are divided into five groups: low, low-middle, middle, high-middle, and high SDI groups. By comparing SDI, researchers and policymakers can better understand the development levels and health status of different regions. In GBD 2021, HHD refers to heart disease caused by hypertension, including heart failure due to impaired or preserved left ventricular function. The GBD network uses standardized tools based on Bayesian methods to integrate data across age, time, region, and health domains, filling gaps in countries lacking primary data sources and enabling global estimates of HHD burden. All GBD estimates include a 95% uncertainty interval (UI), calculated from the 25th and 975th ordered values of the posterior distribution. For countries with sparse data, the interval is wider, indicating relatively lower estimation accuracy.

In the GBD 2021 study, daily temperature data for each location is sourced from the European Centre for Medium-Range Weather Forecasts. The Theoretical Minimum Risk Exposure Level (TMREL) represents the temperature linked with the lowest mortality risk at a specific location and year, adjusting for different disease combinations included in the assessment. Since TMREL often varies between warm and cold regions and changes over time, GBD 2021 utilizes a flexible TMREL that adapts by location and year, rather than a fixed global value (20). Exposure to non-optimal temperature is identified as environmental temperatures that deviate above or below TMREL on any given day, classifying high-temperature exposure as above TMREL and low-temperature exposure as below it. The Population Attributable Fraction (PAF) quantifies the proportion of disease or death reduction achievable if exposure to a risk factor is minimized to the TMREL (i.e., the proportion of cause-specific mortality attributable to daily high or low temperature exposure). The PAF for non-optimal temperature is calculated by combining high- and low-temperature PAFs for each location and year. The PAF for continuous risk exposure across age, gender, location, and year can be calculated using the following formula (Equation 1):


(1) $$PAF=\frac{\sum_{i=1}^{n}P_{i}\left(RR_{i}-1\right)}{\sum_{i=1}^{n}P_{i}\left(RR_{i}-1\right)-1}$$

Here, Pi indicates the percentage of the population exposed to the i-th level of high or low temperature, and n is the total number of exposure levels. The relative risk RRi reflects the risk for exposure at the i-th level of high or low temperature, derived from 81 published systematic reviews. Attributable deaths (ADs) are determined by multiplying the PAF by the count of HHD deaths (N) (21). This can be represented as Equation 2:


(2) AD=PAF∗N

This study focuses on the mortality and DALYs from hypertensive heart disease (HHD) attributed to non-optimal temperature among the older adults aged 60 and above. We extracted data on mortality and DALYs due to hypertensive heart disease (HHD) from low, high, and non-optimal temperatures from the Global Health Data Exchange (GHDx, http://ghdx.healthdata.org/gbd-results-tool) for the years 1990 to 2021, stratified by location, cause, risk factor, age, and gender. The main burden metrics in this study include mortality rate (MR) from hypertensive heart disease due to non-optimal temperature, DALYs, age-standardized mortality rate (ASMR), and age-standardized DALYs (ASDR). The GBD study utilized de-identified datasets, and with the approval of the University of Washington Institutional Review Board, informed consent was waived, thus requiring no additional ethical approval.

### 2.2 Statistical analysis

We used the following linear regression to evaluate trends in ASRM and ASDR related to cold and heat for HHD among older adults populations globally, regionally, and nationally from 1990 to 2021, and estimated the EAPC (Equations 3, 4):


(3) y=α+βx+ε

(4) $$\mathrm{EAPC}=100*\left(\exp\left(\beta \right)-1\right)$$

In this formula, y represents ln(ASRM) and ln(ASDR); x represents the calendar year; and *β* indicates the annual average change in ASRM and ASDR caused by low, high, or non-optimal temperatures. When the EAPC and its lower confidence interval (CI) limit are both greater than 0, it is assumed that the trends in ASRM and ASDR are increasing. When the EAPC and the upper limit of its CI are both less than 0, it is assumed that the EAPC shows a downward trend. Other cases are considered to represent a stable trend. In addition, based on data from 204 regions, Pearson correlation analysis was conducted to examine the correlation between SDI scores and ASMR, ASDR, as well as their corresponding EAPC values.

The age-period-cohort (APC) model is a common statistical tool primarily used to analyze hidden information in mortality, including assessing the mortality risk of populations in specific years and the cumulative health risks from birth. This model can independently assess the influence of age, period, and cohort on cardiovascular disease mortality trends and is widely applied in epidemiological studies of chronic diseases (22). The age effect refers to risk differences across different age groups (23). The period effect reflects changes in cardiovascular disease mortality across age groups under social, cultural, economic, or natural environmental shifts. The birth cohort effect accounts for early life risk factors and environmental conditions of individuals born in the same year. In our analysis, mortality and population data were divided into five-year age groups from 60–64 to 95–99, five-year period groups from 1992 to 2021, and five-year birth cohort groups from 1897–1901 to 1957–1961. Due to the issue of complete collinearity, parameter coefficients were calculated using the intrinsic estimator method, with their exponential form exp(coef) representing the relative risk (RR) of specific ages, periods, or birth cohorts.

In this study, we employed a Bayesian age-period-cohort (BAPC) model, using integrated nested Laplace approximations (INLA) to project sex-specific age-standardized mortality rates (ASMR) and age-standardized disability-adjusted life years (ASDR) due to high and low temperatures in the older adults population to 2050. Studies show that the BAPC model has a high coverage rate and low error rate, making it suitable for robust predictive analyses. Detailed methodologies for the BAPC model have been outlined in relevant literature (24).

All analyses were conducted in R software (version 4.3.2), with data cleaning and organization handled using the tidyverse and dplyr packages. Packages used for ASMR and ASDR prediction included BAPC, nordpred, and INLA. Data visualization was performed with the ggplot2 package. Analysis of the APC model was conducted using STATA 16.0 software, with the significance of parameters and functions assessed via Wald chi-square tests, and all statistical tests were two-sided with a significance level of *p* < 0.05.

## 3 Result

### 3.1 Global and regional burden of hypertensive heart disease in the older adults attributable to non-optimal temperatures, 1990–2021

**Figure 1:**
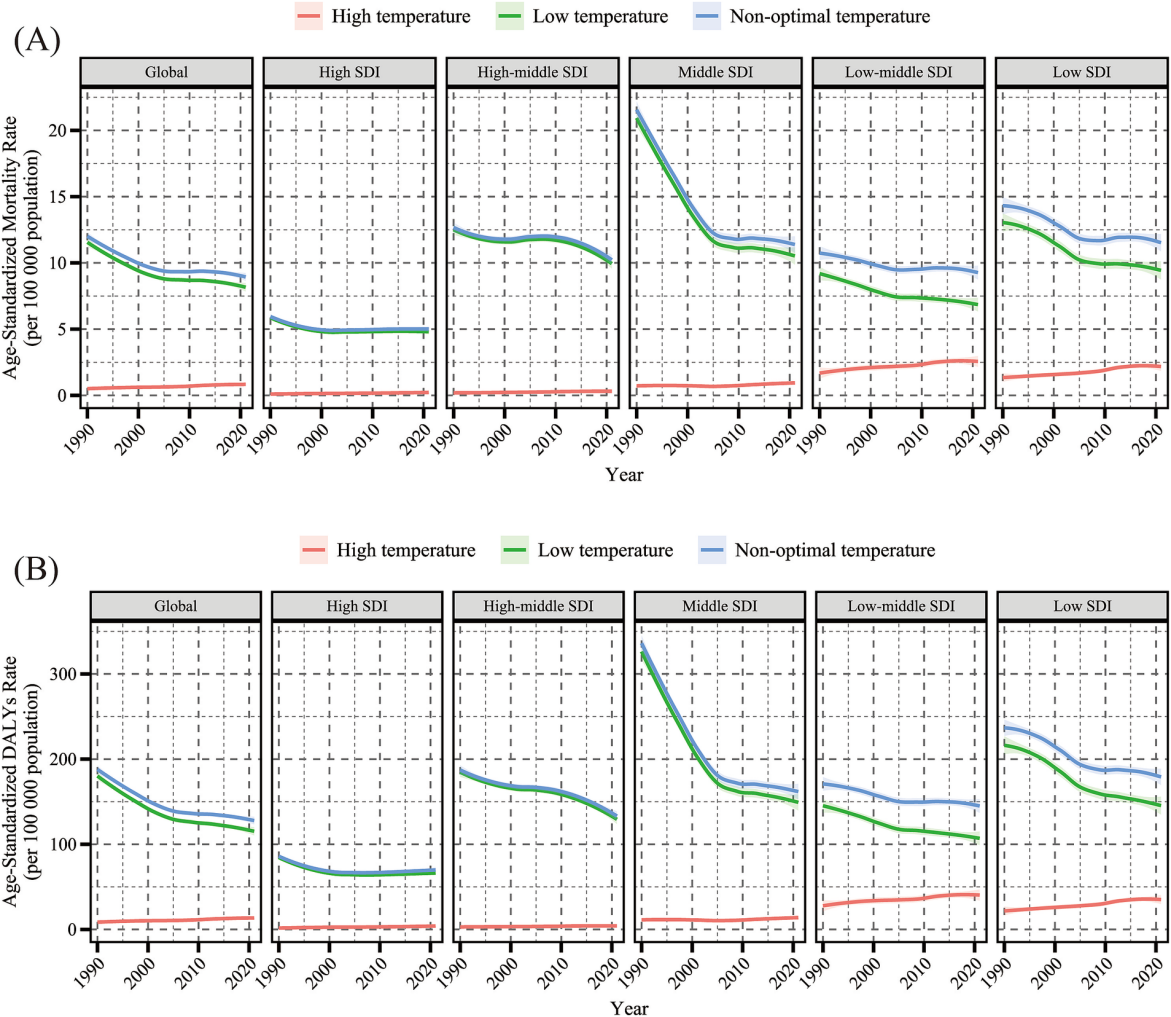
Temporal trends in age-standardized mortality rate (per 100,000 population) **(A)** and age-standardized DALYs rate (per 100,000 population) **(B)** for hypertensive heart disease in the older adults population attributable to low, high, and non-optimal temperatures globally and in five SDI regions, 1990–2021. DALYs, disability-adjusted life years; SDI, sociodemographic index. Shaded areas represent 95% uncertainty intervals.

**Figure 4:**
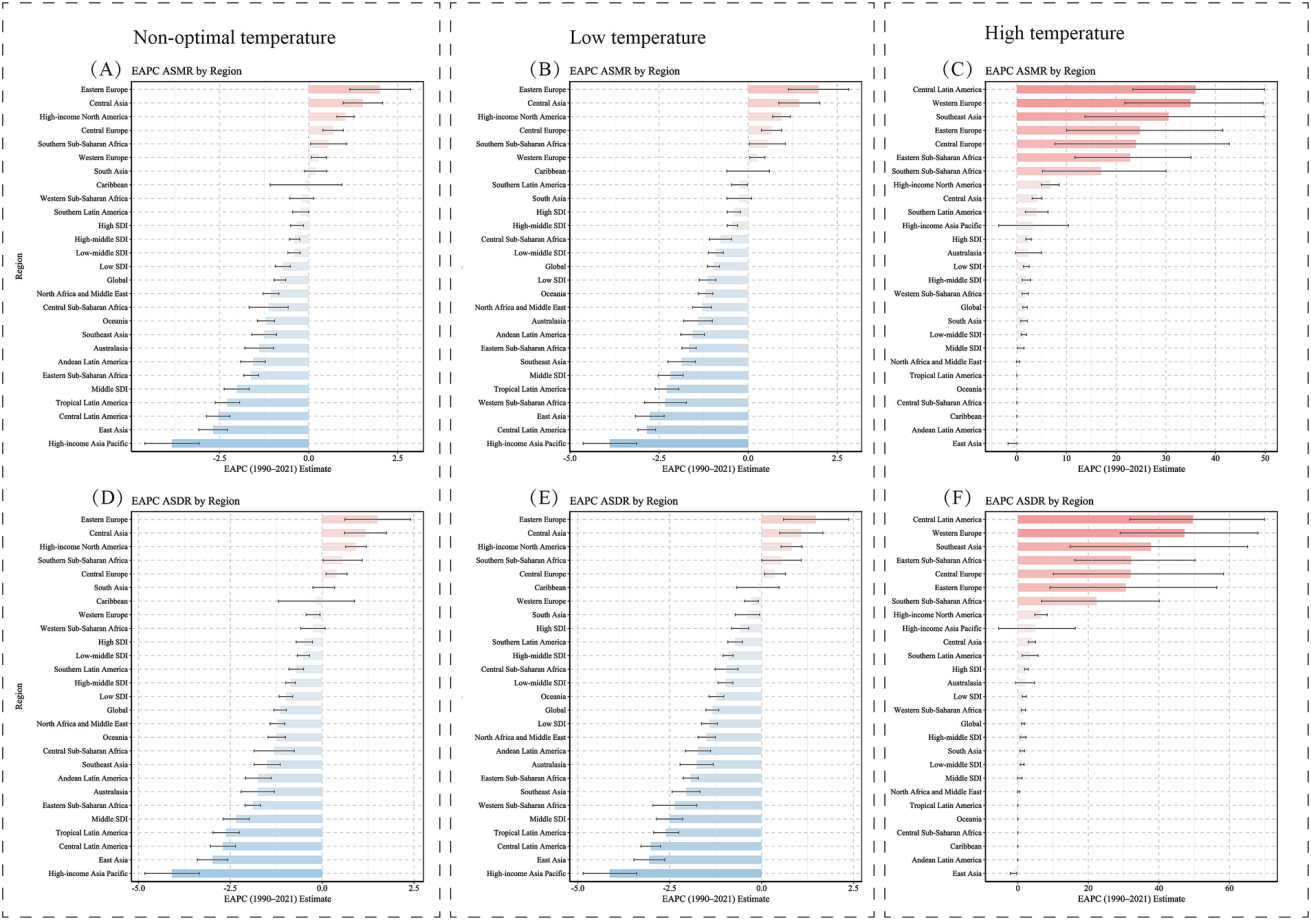
Regional EAPC in ASMR and ASDR (per 100,000 population) for hypertensive heart disease in the older adults population attributable to non-optimal temperature, low temperature and high temperature (1990–2021). EAPC, Estimated Annual Percentage Change; ASMR, Age-Standardized Mortality Rate; ASDR, Age-Standardized DALY Rate; DALYs, disability-adjusted life years.

Globally, during the period from 1990 to 2021, the cold-related burden of HHD in the older adults far exceeded the heat-related burden (Figure 1). However, from 1990 to 2021, the cold-related ASMR of HHD in the older adults population worldwide (per 100,000) decreased from 11.04 (95% UI: 8.79, 12.65) to 8.1 (6.47, 9.56), with an EAPC of −0.97% (95% CI: −1.14, −0.81%). The ASDR (per 100,000) also significantly dropped from 171.85 (134.66, 197.71) to 114.15 (91.84, 134.64), with an EAPC of −1.14% (−1.31, −0.97%) (Figures 4B,E; Supplementary Table S1). Conversely, the heat-related ASMR of HHD in the older adults population increased from 0.55 (−0.66, 2.11) to 0.75 (−0.48, 2.31), with an EAPC of 1.65% (1.18, 2.11%). The ASDR also rose significantly from 9.12 (−10.28, 34.57) to 12.14 (−6.74, 36.08), with an EAPC of 1.46% (1, 1.93%) (Figures 4C,F; Supplementary Table S1).

By SDI region stratification, in 2021, the ASMR [10.39 (7.09, 13.28)] and ASDR [147.31 (101.18, 187.32)] for cold-related HHD were highest in high-middle and middle SDI regions. The ASMR [2.21 (−0.29, 5.45)] and ASDR [34.89 (−4.47, 85.94)] for heat-related HHD were highest in low-middle and low SDI regions. From 1990 to 2021, the cold-related ASMR and ASDR in the older adults population showed a decreasing trend across all five SDI regions, with the lowest EAPC in the middle SDI region. In contrast, the heat-related ASMR and ASDR in the older adults population exhibited an increasing trend in all SDI regions except the high SDI region, where the EAPC was the highest (Figures 1, 4; Supplementary Table S1).

**Figure 2:**
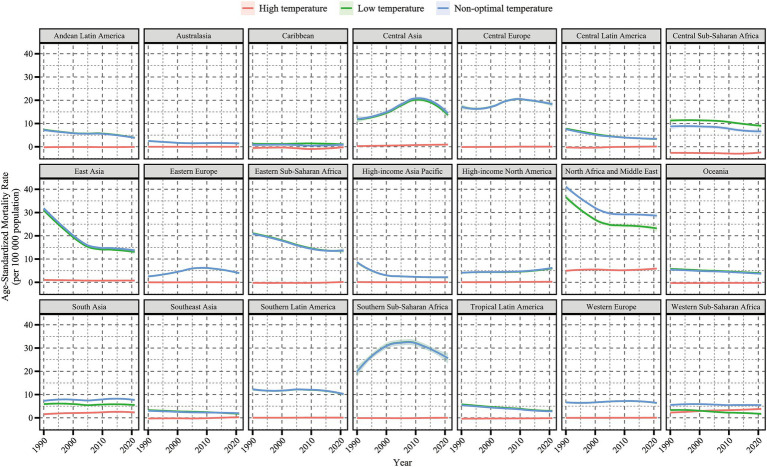
Temporal trends in age-standardized mortality rate (per 100,000 population) for hypertensive heart disease in the older adults population attributable to low, high, and non-optimal temperatures globally and in 21 regions, 1990–2021. Shaded areas represent 95% uncertainty intervals.

**Figure 3:**
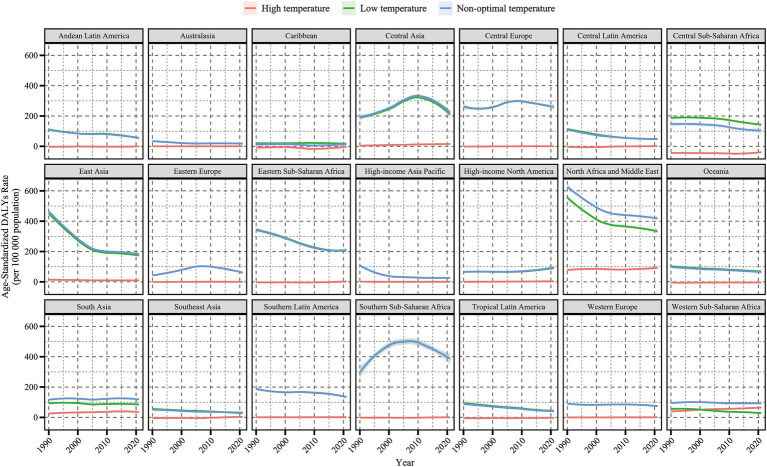
Temporal trends in age-standardized DALYs rate (per 100,000 population) for hypertensive heart disease in the older adults population attributable to low, high, and non-optimal temperatures globally and in 21 regions, 1990–2021. DALYs, disability-adjusted life years; shaded areas represent 95% uncertainty intervals.

By geographic region, in 2021, Central Europe, Eastern Sub-Saharan Africa, and Southern Sub-Saharan Africa had the highest cold-related ASMR [27.39 (22.26, 35.05)] and ASDR [416.84 (342.7, 530.21)] of HHD among the older adults population. North Africa and the Middle East had the highest heat-related ASMR [6.17 (−0.71, 15.06)] and ASDR [96.04 (−9.62, 233.94)] in the older adults population. From 1990 to 2021, among all 21 regions, the cold-related ASMR and ASDR of HHD in the older adults population showed a downward trend in most regions, except in Eastern Europe, Central Asia, and high-income North America (Figures 2, 3). In contrast, the heat-related ASMR and ASDR exhibited an upward trend in most regions except East Asia (Figure 4; Supplementary Table S1).

### 3.2 National burden of hypertensive heart disease in the older adults attributable to non-optimal temperatures, 1990–2021

**Figure 5:**
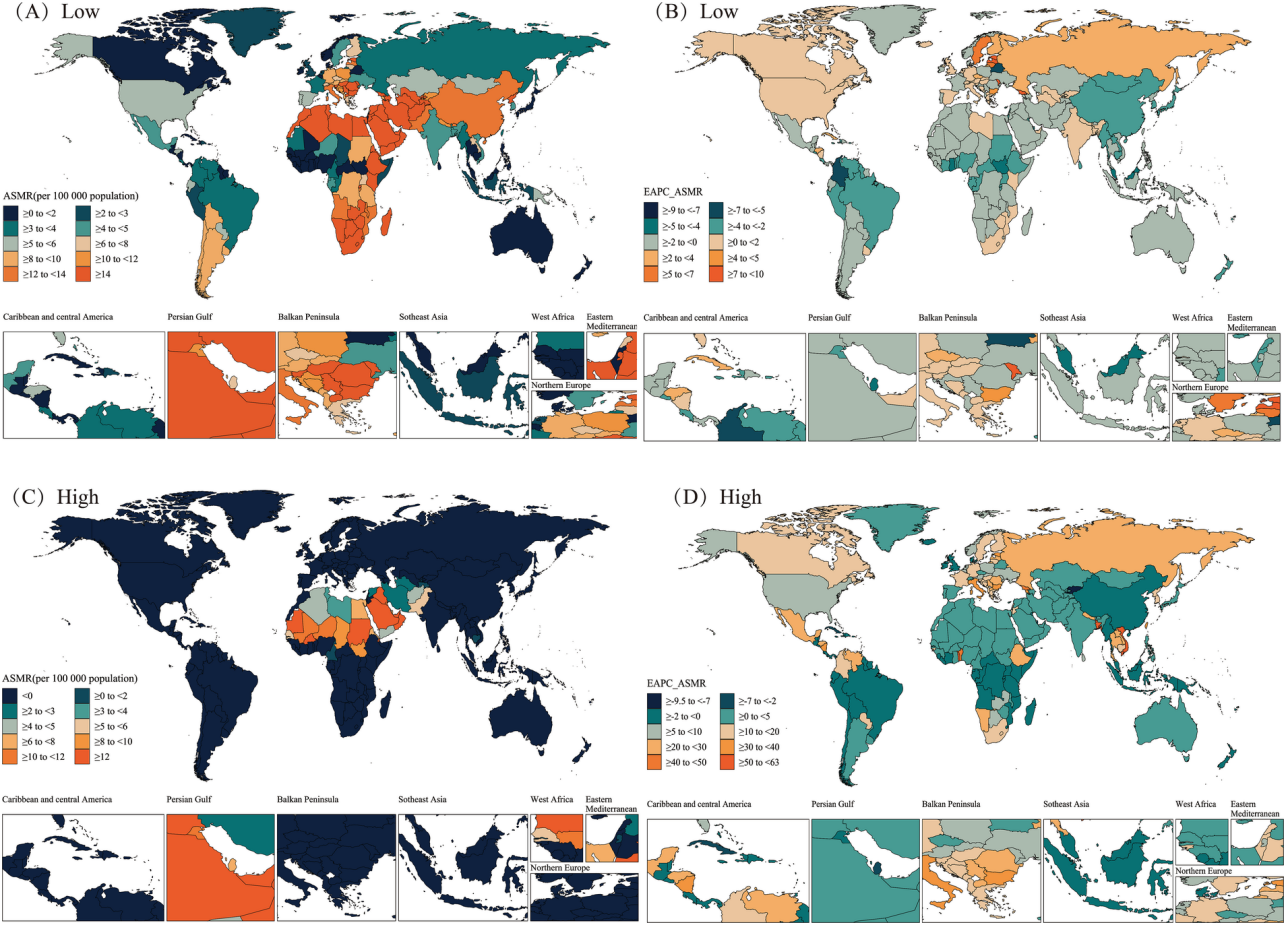
Spatial distributions of ASMR and EAPC in ASMR (per 100,000 population) for hypertensive heart disease in the older adults population attributable to low **(A,B)**, high **(C,D)** temperatures in 2021. ASMR, Age-Standardized Mortality Rate; EAPC, Estimated Annual Percentage Change.

**Figure 6:**
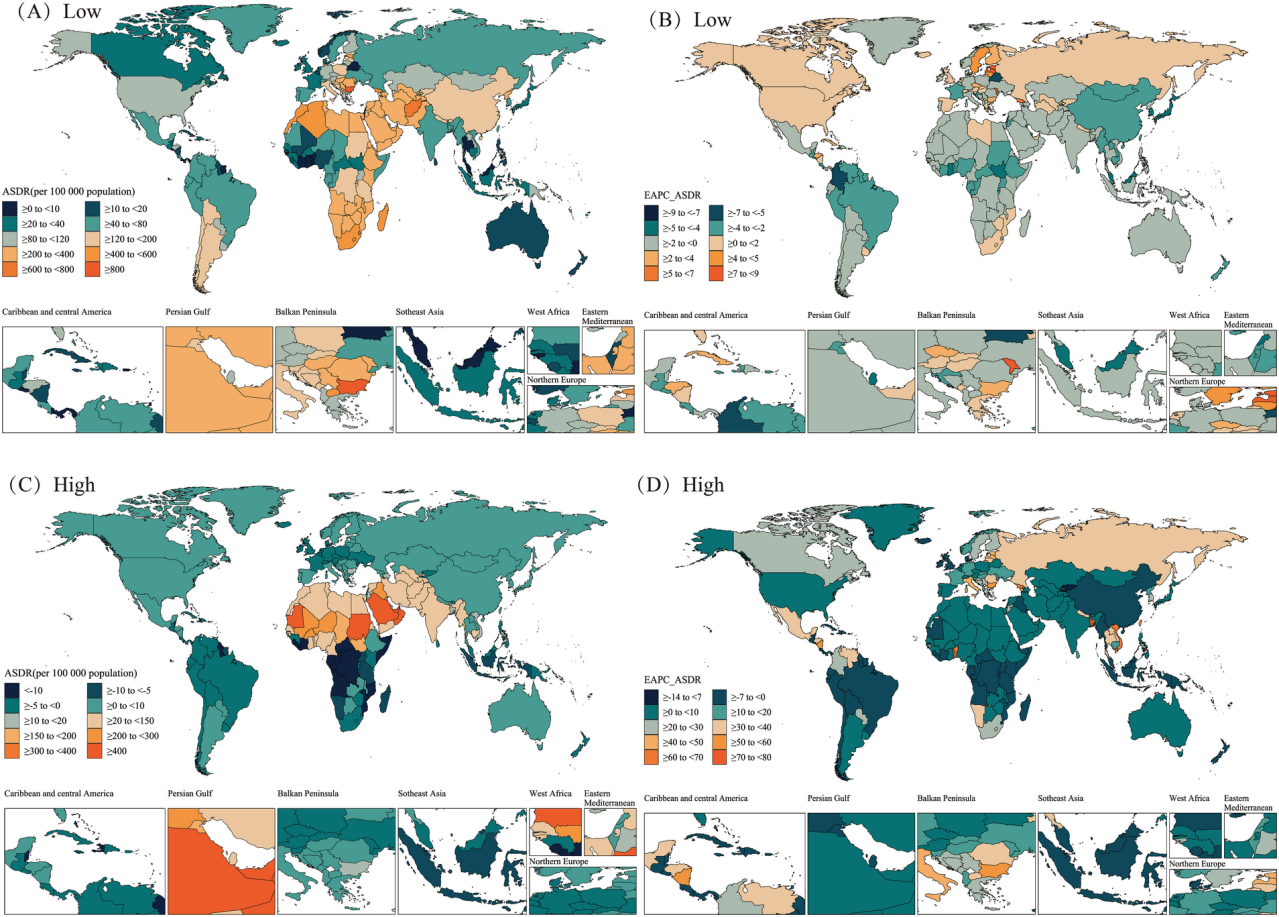
Spatial distributions of ASDR and EAPC in ASDR (per 100,000 population) for hypertensive heart disease in the older adults population attributable to low **(A,B)**, high **(C,D)** temperatures in 2021. ASDR, Age-Standardized DALY Rate; DALYs, disability-adjusted life years; EAPC, Estimated Annual Percentage Change.

At the national level, in 2021, Bulgaria, Lesotho, Afghanistan, and Estonia from the regions of Eastern Europe, Southern Sub-Saharan Africa, South Asia, and Central Europe had the highest cold-related ASMR and ASDR for HHD in the older adults population. Additionally, the United Arab Emirates, Sudan, Oman, Mauritania, and Saudi Arabia—all from the North Africa and Middle East region—exhibited the highest heat-related ASMR and ASDR for older adults HHD (Figures 5, 6).

From 1990 to 2021, most countries/regions exhibited a downward trend in cold-related ASMR and ASDR for older adults HHD, with Guam, Marshall Islands, Micronesia (Federated States of), and Trinidad and Tobago from the High-income Asia Pacific, Oceania, and Latin America and Caribbean—Caribbean regions showing the greatest declines. In contrast, most countries/regions showed an upward trend in heat-related ASMR and ASDR for older adults HHD, with Georgia, Togo, and Viet Nam exhibiting the largest increases. Notably, some countries or regions showed ASMR and ASDR trends that opposed the mainstream trend. Among these, Estonia, Latvia, Republic of Moldova, and Georgia from the regions of Central Europe, Eastern Europe, and Central Asia exhibited the largest increases in cold-related ASMR and ASDR for older adults HHD. In contrast, Qatar, Kuwait, and China exhibited decreases in heat-related ASMR and ASDR for older adults HHD (Figures 5, 6).

**Figure 7:**
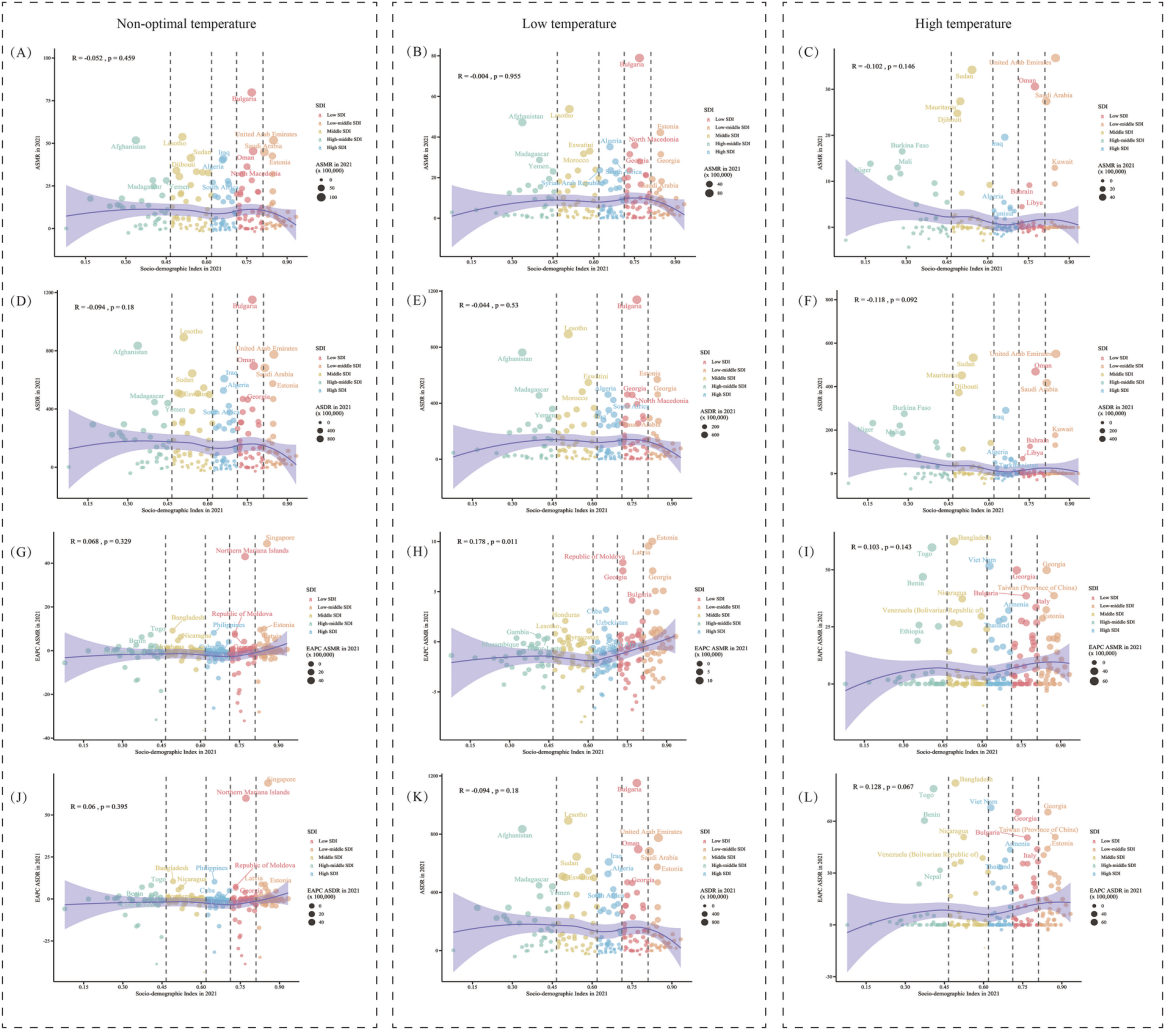
The relationship between SDI and age-standardized rates and their EAPCs for hypertensive heart disease in the older adults. **(A–C)** Show the correlation between SDI and ASMR, while **(D–F)** show the correlation between SDI and ASDR, attributable to non-optimal, low, and high temperatures in 2021. **(G–L)** illustrate the correlation between SDI and the estimated annual percentage change (EAPC) for ASMR and ASDR, respectively. SDI, sociodemographic index; ASMR, Age-Standardized Mortality Rate; ASDR, Age-Standardized DALY Rate; DALYs, disability-adjusted life years; EAPC, Estimated Annual Percentage Change.

At the national level, the correlation between the 2021 SDI score and ASR, as well as EAPC, was analyzed, with the resulting trends shown in Figure 7. Overall, the trends of ASMR and ASDR with changes in SDI scores were quite similar, both showing a slight downward trend. However, the EAPC values of ASMR and ASDR showed an opposite trend with SDI score changes, with an upward trend as SDI increased. Notably, the EAPC value for cold-related ASDR burden showed a declining trend as SDI scores increased.

### 3.3 Age and sex-specific analysis of hypertensive heart disease burden in the older adults attributable to non-optimal temperatures

**Figure 8:**
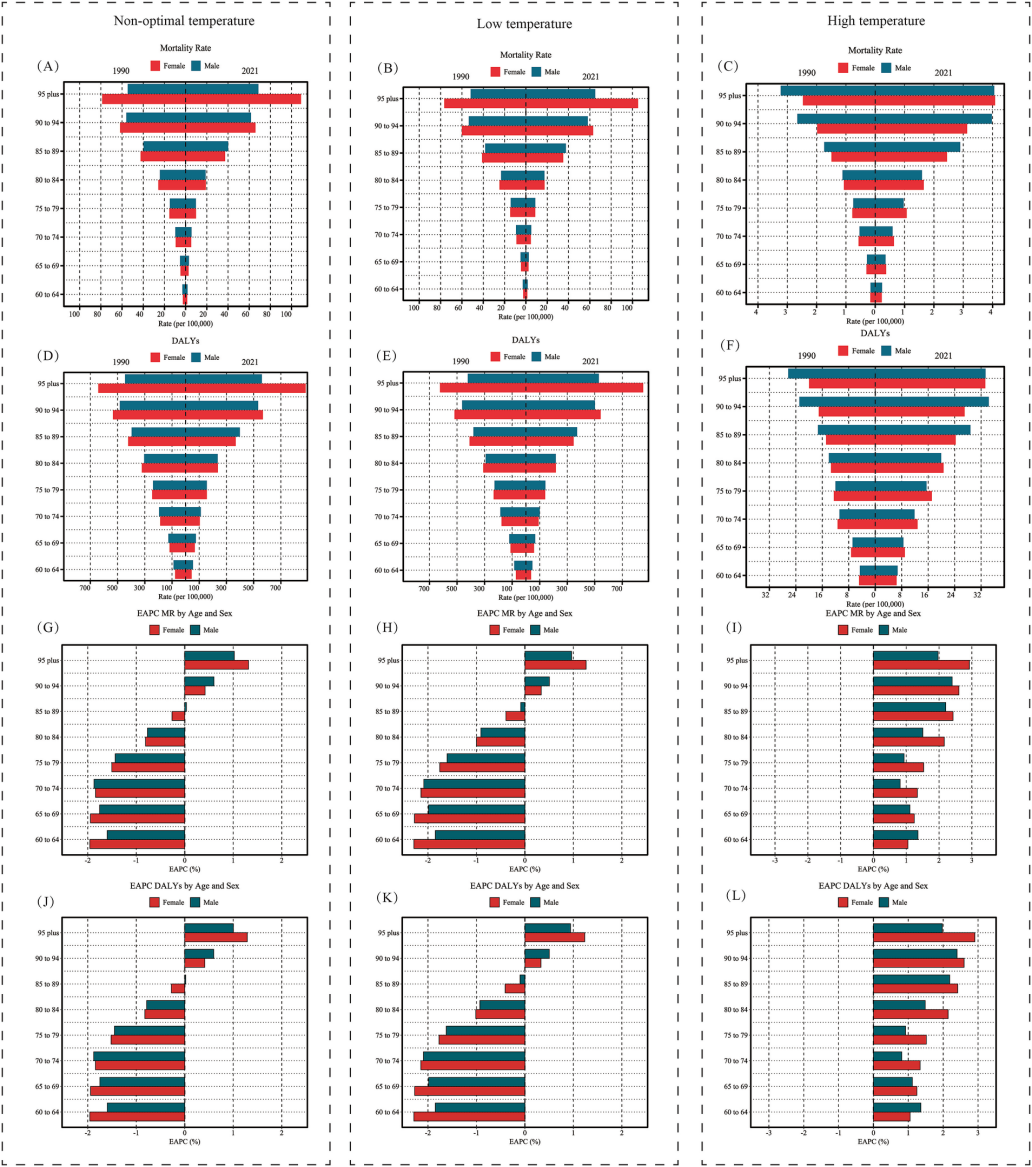
Age- and sex-specific trends in hypertensive heart disease burden attributable to non-optimal, low, and high temperatures in the older adults population, 1990 and 2021. **(A–C)** Display the age-specific mortality rate (per 100,000 population) for males and females in 1990 and 2021, attributable to non-optimal, low, and high temperatures, respectively. **(D–F)** Show the age-specific DALYs rate (per 100,000 population) for the same years and temperature conditions. **(G–I)** Present the EAPC in mortality rate by age and sex, illustrating changes in mortality rate trends from 1990 to 2021. **(J–L)** Depict the EAPC in DALYs by age and sex, showing the trends in DALYs rates over the same period. Red bars represent female rates, while blue bars represent male rates. DALYs, disability-adjusted life years; EAPC, Estimated Annual Percentage Change.

Globally, from 1990 to 2021, the burden of HHD caused by low temperatures in the older adults population exhibited a trend of gradual increase with advancing age across most age groups (Figure 8). In the burden caused by low temperatures, women generally faced a higher burden than men; however, in relatively younger age groups, the burden was slightly higher for men than for women (Figures 8B,E). Calculating EAPC revealed a downward trend in low-temperature-related HHD burden among the older adults population below 90 years from 1990 to 2021, while the burden increased in age groups above 90 (Figures 8H,K). Contrary to the burden caused by low temperatures, in the burden associated with high temperatures, men generally faced a greater burden, although women may experience a higher burden in relatively younger age groups (< 85 years). Notably, among the older adults population, the high-temperature burden showed an increasing trend from 1990 to 2021, with EAPC values greater than 0 across all age groups (Figures 8I,L).

### 3.4 Age-period-cohort analysis

**Figure 9:**
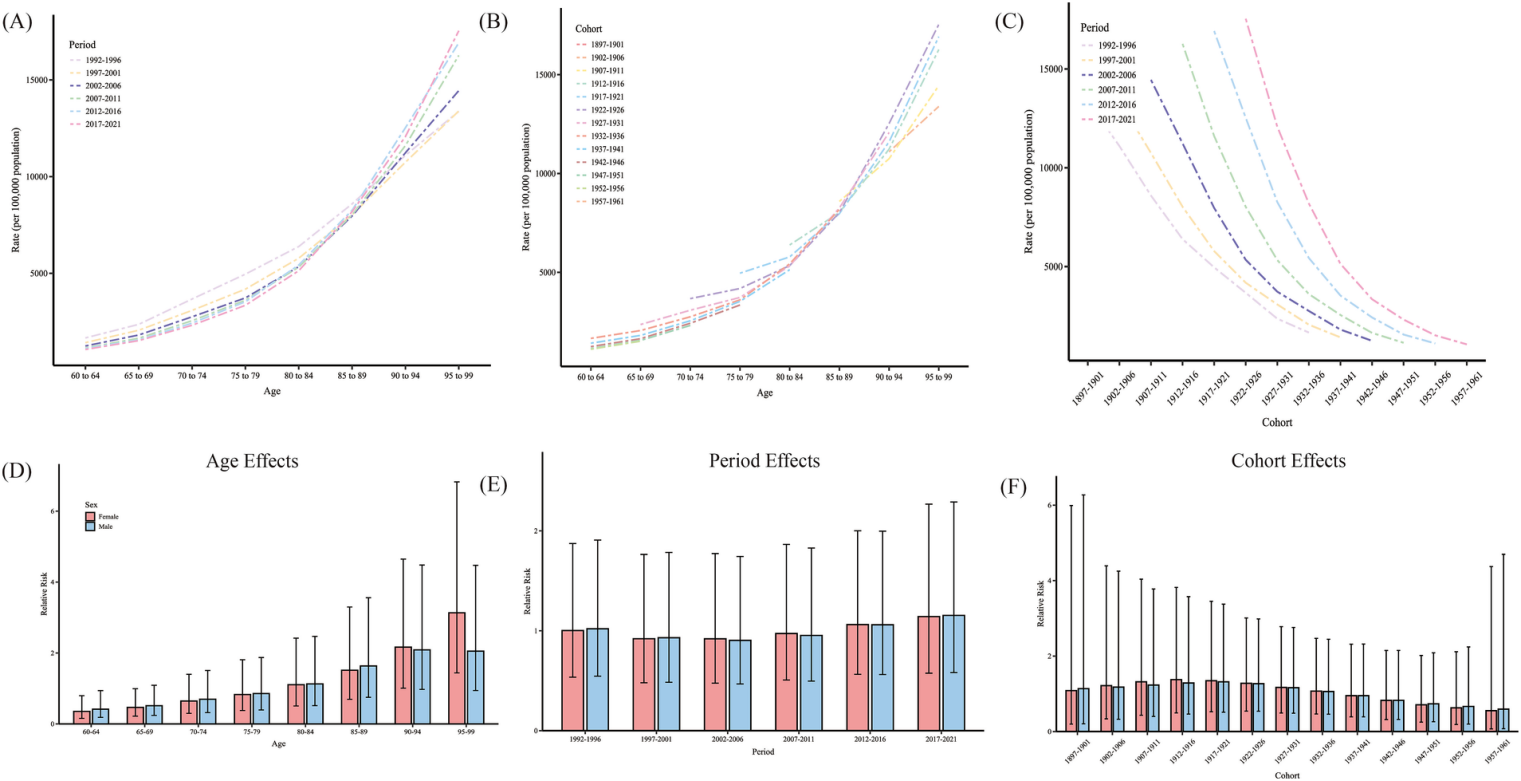
Age-period-cohort analysis of HHD burden attributable to non-optimal temperatures in the older adults population. **(A)** Age-specific HHD rates (per 100,000 population) across different periods from 1992–2021. **(B)** Age-specific HHD rates across different birth cohorts. **(C)** Cohort-specific HHD rates across different time periods. **(D)** Relative risk (RR) of HHD by age for females (red) and males (blue). **(E)** Relative risk of HHD by period for females and males. **(F)** Relative risk of HHD by birth cohort for females and males. Error bars represent 95% confidence intervals for each estimate. HHD, hypertensive heart disease.

Birth cohorts were generated from the data to comprehensively analyze non-optimal temperature effects across age, period, and cohort dimensions. Grouped by time periods, incidence rates increased significantly with age, especially in the older adults (85+), with sharply rising incidence rates; curves in different periods showed similar trends. At age 85, period curves intersected, with burden decreasing as time periods progressed below age 85 but increasing beyond age 85 (Figure 9A). The age burden trends, stratified by different birth cohorts, were similar across cohorts, with disease burden rising significantly with age. However, for earlier birth cohorts, the burden at higher ages was slightly lower than that of later-born cohorts, suggesting a potential increase in disease burden in subsequent cohorts (Figure 9B). The incidence rates by cohort, divided by time periods, showed that earlier-born cohorts (e.g., 1897–1901) had a higher burden, while later cohorts exhibited a declining trend, especially noticeable in more recent periods (2017–2021) (Figure 9C).

Due to collinearity among age, period, and cohort, directly assessing net effects is challenging, and the APC model can be used to address this issue. The age, period, and cohort effects of non-optimal temperature on HHD mortality rates for men and women are shown in Figure 9 and Table 1. After adjusting for bias, the effect of age on female HHD increased from 0.35 (0.16, 0.80) at 60–64 years to 3.13 (1.44, 6.83) at age 95 and older. For men, the age effect increased from 0.42 (0.18, 0.94) at 60–64 years to 2.05 (0.94, 4.47) at 95 and older. The risk of mortality burden varied modestly across different periods and cohorts, with no significant period or cohort effects observed.

**Table 1:** Sex-specific relative risks of hypertensive heart disease death in the older adults population attributable to non-optimal temperatures globally, analyzed by age, period, and cohort effects.

Age	RR (95%)	P	RR (95%)	P
age_60	0.35 (0.16, 0.80)	0.012	0.42 (0.18, 0.94)	0.035
age_65	0.47 (0.22, 0.99)	0.048	0.51 (0.24, 1.09)	0.082
age_70	0.65 (0.30, 1.40)	0.267	0.70 (0.32, 1.51)	0.356
age_75	0.83 (0.38, 1.81)	0.637	0.86 (0.39, 1.87)	0.699
age_80	1.11 (0.51, 2.42)	0.801	1.13 (0.51, 2.47)	0.764
age_85	1.51 (0.70, 3.30)	0.296	1.63 (0.75, 3.56)	0.217
age_90	2.17 (1.01, 4.65)	0.047	2.09 (0.97, 4.48)	0.059
age_95	3.13 (1.44, 6.83)	0.004	2.05 (0.94, 4.47)	0.04
Period				
period_1992	1.00 (0.54, 1.87)	0.795	1.02 (0.55, 1.91)	0.75
period_1997	0.92 (0.48, 1.77)	0.803	0.93 (0.49, 1.78)	0.828
period_2002	0.92 (0.48, 1.77)	0.801	0.90 (0.47, 1.74)	0.762
period_2007	0.97 (0.51, 1.86)	0.733	0.95 (0.50, 1.83)	0.886
period_2012	1.06 (0.56, 2.00)	0.652	1.06 (0.56, 2.00)	0.857
period_2017	1.14 (0.58, 2.27)	0.704	1.15 (0.58, 2.29)	0.682
Cohort				
cohort_1897	1.09 (0.20, 5.99)	0.725	1.14 (0.21, 6.27)	0.883
cohort_1902	1.22 (0.34, 4.39)	0.766	1.18 (0.33, 4.25)	0.804
cohort_1907	1.32 (0.43, 4.04)	0.628	1.23 (0.40, 3.78)	0.714
cohort_1912	1.38 (0.50, 3.82)	0.54	1.29 (0.46, 3.57)	0.627
cohort_1917	1.35 (0.53, 3.45)	0.536	1.32 (0.51, 3.38)	0.567
cohort_1922	1.28 (0.54, 3.01)	0.574	1.27 (0.54, 2.99)	0.587
cohort_1927	1.17 (0.49, 2.78)	0.725	1.16 (0.49, 2.76)	0.737
cohort_1932	1.07 (0.47, 2.47)	0.87	1.06 (0.46, 2.45)	0.89
cohort_1937	0.95 (0.39, 2.32)	0.709	0.95 (0.39, 2.32)	0.51
cohort_1942	0.83 (0.32, 2.15)	0.697	0.83 (0.32, 2.15)	0.698
cohort_1947	0.71 (0.25, 2.02)	0.519	0.74 (0.26, 2.09)	0.564
cohort_1952	0.63 (0.19, 2.12)	0.453	0.67 (0.20, 2.24)	0.512
cohort_1957	0.55 (0.07, 4.37)	0.575	0.59 (0.08, 4.70)	0.622
Deviance	0.01		0.02	
AIC	31.06		30.13	
BIC	−92.87		−92.89	

RR denotes the relative risk of death in particular age, period, or birth cohort relative to the average level of all ages, periods, or birth cohorts combined. RR, relative risk; CI, confidence interval; AIC, Akaike Information Criterion; BIC, Bayesian Information Criterion.

### 3.5 Global gender-specific projections of hypertensive heart disease burden in the older adults due to low and high temperatures from 2022 to 2050

**Figure 10:**
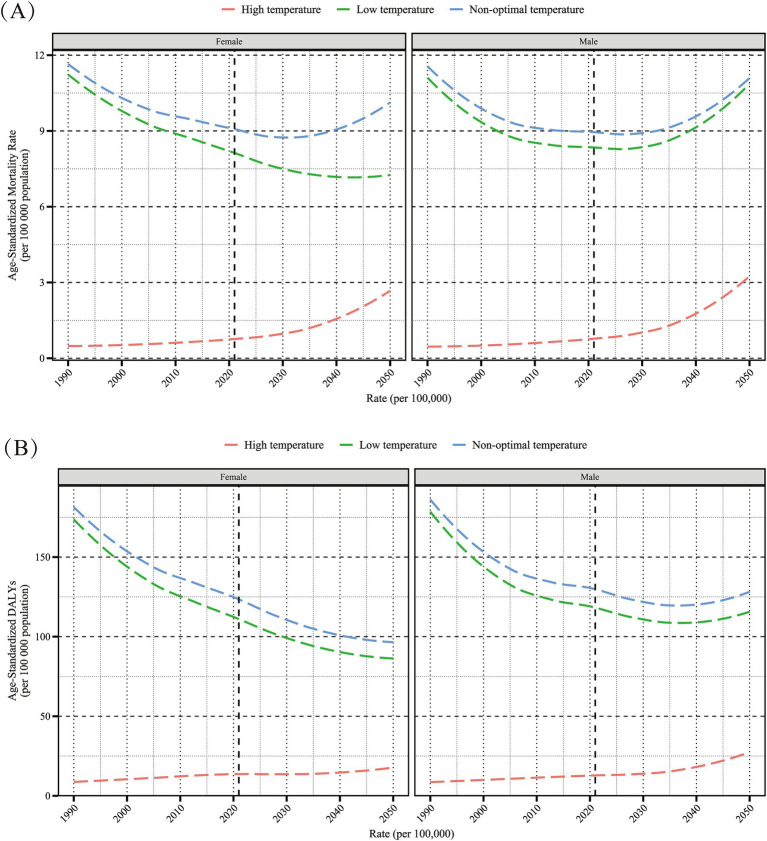
Projected sex-specific trends in age-standardized mortality rate (per 100,000 population) **(A)** and age-standardized DALYs rate (per 100,000 population) **(B)** for hypertensive heart disease in the older adults population attributable to low, high, and non-optimal temperatures, 2022–2050. DALYs, disability-adjusted life years.

The BAPC forecast predicts a continued decline in ASDR burden of HHD due to non-optimal temperature for older adults women from 2022 to 2050, though ASMR is expected to rebound after a brief decrease starting around 2030 (Figure 10). Among older adults men, the non-optimal temperature-induced ASDR burden of HHD is expected to decline until around 2035, followed by a rebound; ASMR is projected to have a shorter decreasing trend and a sustained increase. For the HHD burden due to low temperatures, female ASMR and ASDR are forecasted to continue decreasing, whereas male ASMR is projected to increase, with ASDR declining but rebounding around 2035, remaining below current levels overall. The burden of older adults HHD due to high temperatures is projected to show a consistent upward trend across all assessment indicators and both genders.

## 4 Discussion

In this study, we conducted the first spatiotemporal analysis of the burden and trends of HHD associated with abnormal temperatures in individuals aged 60 and above. Overall, the burden of non-optimal temperature on HHD was mainly caused by low temperatures, while the impact of high temperatures was comparatively smaller. From 1990 to 2021, the cold-related HHD burden in the older adults decreased significantly, while the heat-related burden increased markedly, especially in low-income and tropical regions. Among the older adults, the disease burden increased with age, indicating the sensitivity of older adults HHD to temperature changes and the health vulnerability of this age group. Additionally, we found that among the older adults, women’s HHD burden was more sensitive to low temperatures, while men’s HHD burden was more sensitive to high temperatures. Using 85 years as an approximate threshold, the gender gap in HHD burden caused by non-optimal temperatures was smaller before age 85. Although low temperatures will remain the main driver of non-optimal temperature-related HHD burden among the older adults in the coming years, high temperatures are expected to catch up rapidly, underscoring the need for measures to address both factors. Our projections show that the burden of HHD due to all non-optimal temperatures will continue to increase in older adults men, while low-temperature-related HHD burden will decrease in older adults women, but high-temperature burden will continue to rise.

Previous studies generally indicate that low temperatures have a greater adverse impact on health than high temperatures. Consistent with this, we observed that the disease burden of HHD due to low temperatures in the older adults is far greater than that due to high temperatures (25–27). There are several possible explanations for this phenomenon. First, cold weather generally lasts longer, so people are often exposed to low temperatures for a longer period than to high temperatures (28). Second, the rise in global temperatures is still relatively small, with annual average temperatures changing slowly, resulting in a lower burden of cardiovascular disease from high temperatures. However, since the 20th century, global temperatures have risen by approximately 1.25°C, and at the current rate of emissions, they may exceed 1.5°C within the next decade (29). This suggests that future climate warming may further increase the negative impact of high temperatures on health, exacerbating the disease burden associated with high temperatures. Additionally, research has shown that compared to low-temperature exposure, high-temperature exposure is more likely to increase the risk of HHD (30). This may also explain the observed gradual increase in HHD mortality burden from high temperatures among older adults men and women since 1990. Overall, in the context of global warming, we should not only focus on the impact of low temperatures on cardiovascular health but also develop more effective measures to address the potential increase in HHD burden due to high temperatures in the future.

Our study also found a significant gender difference in the burden of HHD caused by non-optimal temperatures. At high temperatures, the mortality burden among most older adults men was higher than that of women, whereas under low temperatures, the HHD burden was greater among most older adults women compared to men. Additionally, between 1990 and 2021, the decline in age-standardized mortality rates EAPC due to heat-induced HHD was generally greater among older adults women compared to men. Previous studies have also indicated that the risk of cardiovascular mortality associated with temperature changes varies by gender, though specific patterns differ by study, with some showing similar risks for both genders, while others report higher risks in either men or women (31–33). The exact cause of this gender disparity is currently unclear, and future prospective studies may help to further elucidate this phenomenon. Some studies hypothesize that this difference may be related to mechanisms of temperature regulation, physiological structure, and sociocultural factors (34, 35). For instance, as women enter menopause, the drop in estrogen levels may enhance vascular constriction in response to cold, thereby increasing the burden of HHD from low temperatures. This physiological difference may partly explain the gender differences observed in our data analysis.

Age-period-cohort analysis indicates that age is a significant risk factor for the burden of HHD among the older adults caused by non-optimal temperatures. As age increases, the impact of temperature on older adults men and women significantly intensifies. This trend aligns with previous studies, most of which indicate that older adults are more susceptible to temperature changes than younger people (36). This phenomenon may be related to age-related declines in temperature regulation capacity, weakening bodily functions, and increased risk of various chronic diseases (37). Based on these findings, as population aging intensifies, governments and health departments should pay particular attention to the older adults and implement targeted measures to help them adapt to the impact of non-optimal temperatures. Measures could include enhancing the prediction and monitoring of extreme temperature events, promptly issuing public warnings; strengthening community health services, educating residents on coping with non-optimal temperatures, and encouraging practical application of this knowledge.

This study systematically evaluated the global burden of HHD caused by non-optimal temperatures and its spatiotemporal trends in the older adults for the first time, providing a scientific basis for formulating older adults-appropriate interventions. However, this study has certain limitations. Firstly, the GBD 2021 methodology did not account for lagged or cumulative temperature effects, focusing only on short-term exposure effects, which may underestimate the burden of disease associated with non-optimal temperatures. Secondly, as temperature exposure is based on environmental temperatures rather than individual-level exposure, there may be an ecological fallacy. Furthermore, as extreme temperature events become more frequent, their impact on disease burden is increasingly significant. However, the GBD 2021 study lacks data on extreme temperature events, making it difficult for us to assess their impact on older adults HHD. Therefore, it is necessary for future research to examine the impact of climate factors such as heat waves, cold spells, and temperature variability on HHD disease burden in the older adults.

## 5 Conclusion

Despite the fact that from 1990 to 2021 the global burden of HHD mortality and DALYs among the older adults was primarily driven by low temperatures, the burden related to high temperatures has increased significantly over the past 30 years and is expected to continue in the next 20 years. In low-income or tropical regions, the disease burden caused by heat is especially pronounced. Additionally, the burden of HHD among the older adults due to non-optimal temperatures shows significant gender differences, with women being more sensitive to cold and men more affected by heat. Therefore, it is necessary to strengthen the adaptability of vulnerable regions and sensitive populations to effectively address the potential health risks posed by future temperature anomalies.

## Data Availability

The original contributions presented in the study are included in the article/Supplementary material, further inquiries can be directed to the corresponding authors.

## Footnotes

^1^ https://ghdx.healthdata.org/record/ihme-data/global-burden-disease-study-2021-gbd-2021-demographics-1950-2021
